# An optimized digital watermarking algorithm in wavelet domain based on differential evolution for color image

**DOI:** 10.1371/journal.pone.0196306

**Published:** 2018-05-21

**Authors:** Xinchun Cui, Yuying Niu, Xiangwei Zheng, Yingshuai Han

**Affiliations:** 1 School of Information Science and Engineering, Qufu Normal University, Rizhao, Shandong Province, China; 2 Peony District Library of Heze City, Heze, Shandong Province, China; 3 School of Information Science and Engineering, Shandong Normal University, Ji’nan, Shandong Province, China; Northeast Normal University, CHINA

## Abstract

In this paper, a new color watermarking algorithm based on differential evolution is proposed. A color host image is first converted from RGB space to YIQ space, which is more suitable for the human visual system. Then, apply three-level discrete wavelet transformation to luminance component Y and generate four different frequency sub-bands. After that, perform singular value decomposition on these sub-bands. In the watermark embedding process, apply discrete wavelet transformation to a watermark image after the scrambling encryption processing. Our new algorithm uses differential evolution algorithm with adaptive optimization to choose the right scaling factors. Experimental results show that the proposed algorithm has a better performance in terms of invisibility and robustness.

## 1 Introduction

With rapid progress of computer technologies, digital products such as images, audios, videos and database files get extensive dissemination over the Internet. However, when people are enjoying the advantages which brought by multimedia technologies, there are also many undesired issues, such as divulgence, interpolation or forgery of digital works, even commerce use to obtain unlawful interests, which will seriously invade owner’s legal interests of product copyright. Currently, there are rich dual modes to resist these behaviors, which fall into two categories. The first one is establishing the state-level legal systems to protect digital copyright and punish illegal offenders [1, 2]; the other one is seeking new technical methods to protect digital copyright [3–5]. Recently, it has drawn the attention of the researchers to protect multimedia by utilizing digital watermarking technique that has been widely applied in many other areas.

Digital watermarking algorithm mainly has two important properties: invisibility and robustness. However, there are certain conflict between them. On the one hand, the smaller value of watermark scaling factors, the better watermarking information invisibility and the weaker algorithm robustness. On the other hand, the larger value of scaling factors, the worse quality of the cover image and the stronger watermarking algorithm robustness. Therefore, how to select suitable scaling factors to balance the relationship between invisibility and robustness, and then improve the performance of the watermarking algorithm is our critical issues. In recent years, a number of optimization algorithms, such as simulated annealing algorithm (SAA), genetic algorithm (GA), particle swarm optimization (PSO), ant colony optimization (ACO), etc, provide some new theories or methods for solving optimization problems, and have been developed gradually by simulating or revealing some certain natural phenomena and process. Pereira et al. proposed a method [6] that considers the limited space domain in a best way and the watermark information embedded as a linear programming problem, expecting to maximize the watermark strength in a series of pixelization linear constraints. Vahedi et al. presented a new color image watermarking algorithm [7], using bionic principles based on wavelet transform which is combined with bionic theory to optimize digital watermarking algorithm.

Differential evolution (DE) algorithm, with strong global searching ability and convergence rate in solving some complex global optimization problems, is an evolutionary scheme based on group differences, which is proved to be an effective global optimum solution technology [8]. In 2009, Aslantas firstly proposed a robust digital watermarking algorithm utilizing differential evolution and singular value decomposition [9], and this scheme verifies that differential evolution can well counterpoise the conflict two competing criteria (invisibility and robustness), and to some extent, enhance its robustness after some common signal processing operations or the geometric attacks. Subsequently, the theory of differential evolution algorithm has been widely used in many fields of digital watermarking [10–15]. Based on the above analysis, a new color watermarking algorithm based on differential evolution in wavelet domain was proposed, which takes color image as cover image and has a comprehensive applicability. This new scheme is different from the traditional scheme, which integrates multi-resolution and local sign analysis characteristics of discrete wavelet transform. It is also based on singular value decomposition theory, under the premise of ensuring better performance of watermarking algorithm. Additionally, this algorithm is secure due to applying Arnold transform on watermark image before embedding progress.

The rest of this paper is organized as follows. In section 2, we review the related preliminaries of this research. Section 3 describes the proposed watermarking scheme in details. The simulation results are analyzed in section 4. Finally, the conclusion is drawn in section 5.

## 2 Preliminaries

### 2.1 Human visual system

The human visual system (HVS), mainly composed by the human eye and visual central nervous system, is an advanced intelligent information processing system. In the digital watermarking technology, HVS is usually involved in the process of image quality assessment. In order to make the effect of objective evaluation method be closer to the subjective assessment, evaluation of the accuracy, monotonic and other aspects will be greatly improved [16]. Under normal circumstances, HVS characteristics mainly include: brightness, edge masking, the texture masked frequency, domain feature and orientation feature [17].

Among them, the brightness feature of HVS is fundamental. Embedding watermark information in the highlight areas will have better invisibility. Color space includes RGB, YIQ, CMY. Yet, RGB format is mainly used for computer display, while YIQ for image communications. Due to the luminance characteristics of the HVS, it is necessary to convert RGB mode image to YIQ color space by color space transformation when embed watermarking information into color images. Compared to the chrominance components I and Q, the human eye is not sensitive to the luminance component Y. Thus, embed the watermarking information into the Y component can strengthen invisibility. Mutual transformation between RGB mode and YIQ format is defined as [18, 19].

$$\begin{array}{c} {}[\begin{array}{c} Y\\ I\\ Q \end{array}]=[\begin{array}{ccc} 0.299 & 0.587 & 0.114\\ 0.596 & -0.275 & -0.321\\ 0.212 & -0.523 & 0.311 \end{array}]\cdot [\begin{array}{c} R\\ G\\ B \end{array}], \end{array}$$ (1)

$$\begin{array}{c} {}[\begin{array}{c} R\\ G\\ B \end{array}]=[\begin{array}{ccc} 1.000 & 0.956 & 0.620\\ 1.000 & -0.272 & -0.647\\ 1.000 & -1.108 & 1.703 \end{array}]\cdot [\begin{array}{c} Y\\ I\\ Q \end{array}]. \end{array}$$ (2)

### 2.2 Arnold transform

The image scrambling is used to disturb the pixel order in an image, which destroys the correlation between the original image pixels and ensures the image transmission security. At present, there are some common transform methods such as Arnold scrambling transform, Fibonacci transform, Hilbert curve, Affine transform, Magic square scrambling, Gray code conversion and Latin squares orthogonal transform. Arnold transform is raised by V.J.Arnold in the study of ergodic theory, commonly known as cat face transform (Arnold’s cat map). Due to its simple calculation, easily implemented and cyclical features, Arnold transform is applied in scrambling processes of watermark image extensively. Arnold transform is defined as follows.

$$\begin{array}{ll} \left[\begin{array}{l} x\prime \\ y\prime \end{array}\right]=\left[\begin{array}{cc} 1 & 1\\ 1 & 2 \end{array}\right]\left[\begin{array}{l} x\\ y \end{array}\right]\;mod(N) & x,\;y\in \left\{0,\;1,\;\ldots ,\;N-1\right\} \end{array},$$ (3)

**Fig 1:**
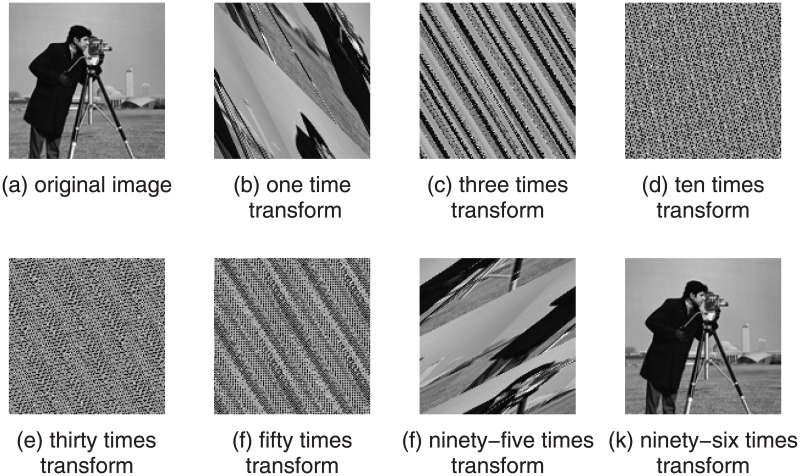
Impression drawing after different Arnold transform times.

Where, (x, y) is the pixel of a original image and (x’, y’) is transformed pixel value of a new image. N is the height or width of square image which is processed. Watermark image through Arnold transform process are shown as in Fig 1. As we can see that the original image is obtained after the conversion cycle T (T = 96) times, so a watermark image will be able to get through the repeated Arnold transform process.

### 2.3 Discrete wavelet transform

**Fig 2:**
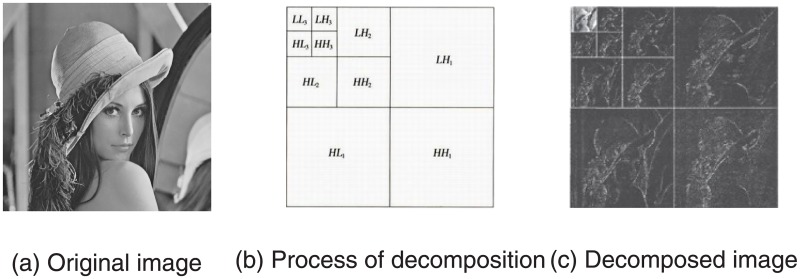
Schematic pictures of three-level discrete wavelet decomposition.

Wavelet transform, a multi-scale signal analysis method, inherits and develops the idea of Fourier transform fixed resolution, having features of multi-resolution, local signal analysis and so forth. It is an ideal tool for frequency analyzing and signal processing [20]. In the past few years, the wavelet transform theory has been widely applied in many related fields, especially the theory of discrete digital algorithm, Discrete Wavelet Transform (DWT). A original image is decomposed into four different space and frequencies sub-bands by DWT, including a low frequency sub-band LL and three high frequency sub-bands HL, LH, HH. Among them LL is the most similar sub-image to the original image, called approximation sub-band. The other three sub-bands LH, HL, HH respectively denote horizontal direction detail, vertical and diagonal detail direction image. Moreover, the low frequency sub-band can be further decomposed into another four different sub-bands. Three-level DWT decomposition is shown in Fig 2. Where, the low frequency component represents the most contents of the original image, and the high frequency represent the edge, contour and texture characteristics of the original image. Fig 2c expresses the different frequency schematic picture of the original image “Lena” and Fig 2a shows the structures of “Lena” image after the three-level discrete wavelet transform decomposition.

### 2.4 Singular value decomposition

Singular value decomposition (SVD) is a matrix diagonalization efficient algorithm in the numerical linear algebra which plays an important role in the matrix theory and calculations. In the image processing community, SVD has the following features [21]:

1. The singular value of an image has the good stability. When a image may be slightly disturbed, its singular values will not change significantly;
2. The first singular value in the value sequence that obtained by SVD operation of an image, is much larger than the rest ones. The reconstructed image quality is not largely degraded, if neglecting these smaller singular values of its item.
3. The singular value demonstrates the inherent algebraic property of a image. The extracted watermark image will always be affected by the process of geometry operations, especially the extraction of blind watermarking algorithm. According to the characteristics of SVD, the cover image is able to withstand certain geometric distortions, if singular value decomposition is performed on the processes of watermark embedding and extraction.

Let A be a rectangular matrix of order, then according to SVD, it can be decomposed mathematically into three matrices as:

$$\begin{array}{c} A=USV^{T}, \end{array}$$ (4)

Where, U and V represent unitary matrix of order *m* and *n*, *UU*^*T*^ = *I*_*m*_, *VV*^*T*^ = *I*_*n*_; *S* is diagonal matrix of order *m* × *n*, the values on the diagonal position are called its singular values of matrix A, represented by *σ*_1_ ≥ *σ*_2_ ≥ … ≥ *σ*_*n*_ ≥ 0 respectively and satisfy the following formula (4). As for the rank of matrix A is *r*(*r* ≤ *n*), A also can be divided as Eq (6).

$$\begin{array}{c} \sigma_{1}\geq \sigma_{2}\geq \ldots \geq \sigma_{r}\geq \sigma_{r+1}\geq \ldots \geq \sigma_{n}=0 \end{array}$$ (5)

$$\begin{array}{c} A=\sum_{i=1}^{r}\sigma_{i}u_{i}v_{i}^{T} \end{array}$$ (6)

Where, *U*_*i*_, *V*_*i*_ are the *k* order eigenvectors of matrices U and V.

### 2.5 Differential evolution algorithm

**Fig 3:**
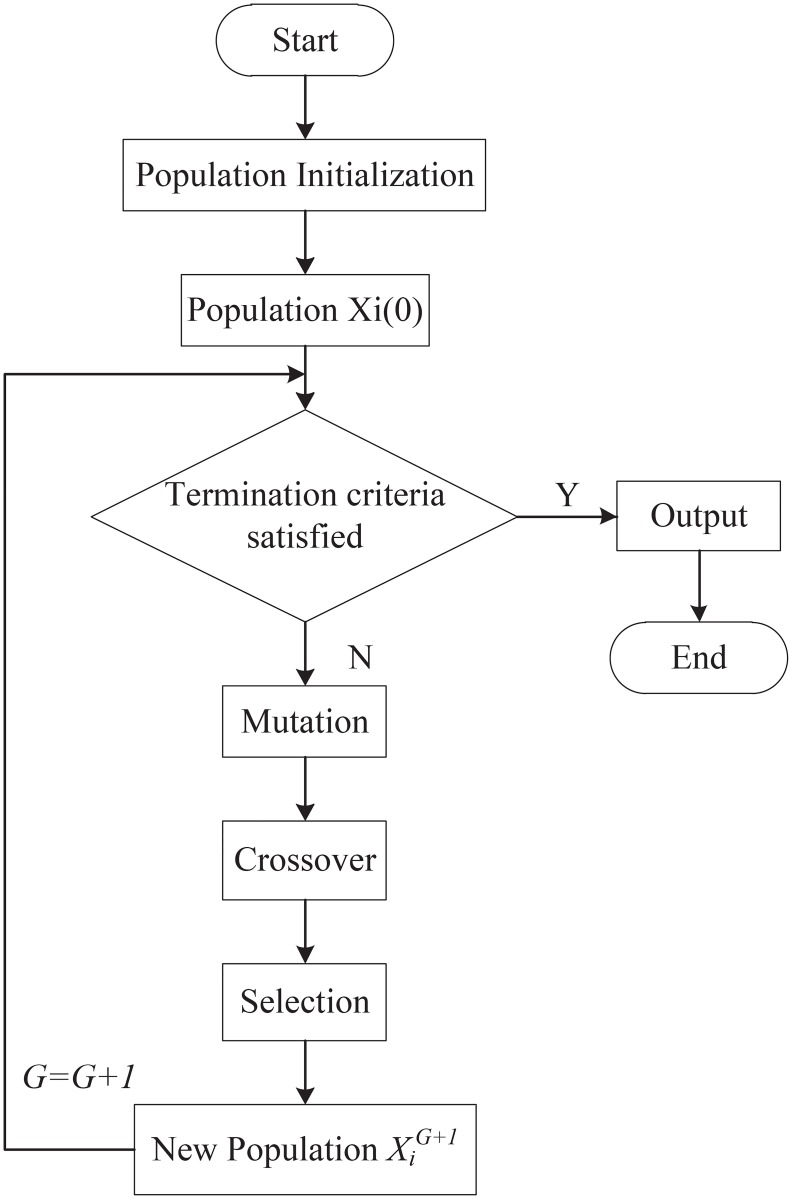
Execute process of differential evolution algorithm.

Differential evolution red (DE) algorithm, a heuristic global search computer technology, was proposed by American scholar Store and Price in 1995, and it was originally intended to solve the problem of chebyshev polynomials earlier [22, 23]. The basic idea of DE algorithm is presented as follows: two different individuals first randomly selected from an initial population are made subtraction to generate a vector difference, and then another individual distinct from the previous ones is chosen to be summed with the vector difference, which obtains a new individual named perturbed one; the perturbed individual then swaps with a target individual which is randomly chosen from the current population according to certain rules, to generate the trial individual in the generated population; the target individual and trial individual finally are competing with each other and the one with the preferred value that is allowed to enter the next generation. Differential evolution algorithm is operated by continuous iteration, generally ameliorating its population quality, which guides the population approaching to the optimal position. Due to DE algorithm properties of simple and efficient theory, easily understand and programming, reliable operation result, strong algorithm robustness and fast convergence etc, this algorithm has been widely applied in many kinds of areas, such as image processing [24], neural networks [25], distribution of economic resources optimization [26], marine engineering [27], and so forth. Execution of the differential evolution algorithm is illustrated in Fig 3, and the following describes the basic strategy of DE algorithm:

1. Initial population
  Determine population size NP each of dimension D, and randomly generate an initial population.
  $$\begin{array}{c} X_{i}\left(0\right)=\left(x_{i,1},x_{i,2},\ldots ,x_{i,D}\right)\;i=1,2,\ldots ,NP \end{array}$$ (7)
  $$\begin{array}{c} x_{i,j}=a_{j}+rand\cdot \left(b_{j}-a_{j}\right)\;j=1,2,\ldots ,D \end{array}$$ (8)
  Where, *X*_*i*_(0) and *x*_*i*,*j*_ are respectively the *i-th* individual of the initial population and the *j-th* individual of the *i-th* component. Rand is random numbers within [0, 1], *a*_*j*_ ≤ *x*_*i*,*j*_ ≤ *b*_*j*_.
2. Mutation
  Mutation operation is the most important step of DE algorithm which name is derived from here. Three distinct individuals $x_{r1}^{G}$, $x_{r2}^{G}$, $x_{r3}^{G}$ (*r*1 ≠ *r*2 ≠ *r*3) are randomly chosen from the current population which generation is G. A differential vector is generated according to the following Eq (9).
  $$\begin{array}{c} D_{r1,2}=x_{r1}-x_{r2} \end{array}$$ (9)
  Then a perturbed individual $V_{i}^{G+1}$ is produced as Eq (10), and the mutation factor *F* ∈ [0, 1] is a real constant.
  $$\begin{array}{c} V_{i}^{G+1}=x_{r3}^{G}+F\left(x_{r1}^{G}-x_{r2}^{G}\right) \end{array}$$ (10)
3. Crossover
  The trial individual $U_{i,j}^{G+1}$ is generated from the crossover operation which is performed between perturbed individual $V_{i}^{G+1}$ that is gotten from the operation of mutation and target individual $x_{i}^{G}$. Mathematically this operation is given as,
  $$\begin{array}{c} U_{i,j}^{G+1}=\left(u_{i,1}^{G},u_{i,2}^{G},\ldots ,u_{i,j}^{G}\right)\;j=1,2,\ldots ,D \end{array}$$ (11)
  $$\begin{array}{c} u_{i,j}^{G+1}=\{\begin{array}{cc} v_{i,j}^{G+1}, & if\;rand\leq CR\;or\;j=j_{rand}\\ x_{i,j}^{G+1}, & \;otherwise \end{array} \end{array}$$ (12)
  Where, the crossover rate constant *CR* ∈ [0, 1] is predefined by the algorithm user and *rand* ∈ [0, 1] is the random number. *j*_*rand*_ ∈ [1, *D*] is a randomly selected integer and the trial individual $U_{i,j}^{G+1}$ represents the *i-th* one that produced from new population of generation G+1. This crossover operation increases the diversity of its population.
4. Selection
  After the mutation and crossover operation, trial individual $u_{i}^{G+1}$ is compared to target individual $x_{i}^{G}$ which is chosen from the original population to decide the best individual that will enter into the next generation, it is defined as follows,
  $$\begin{array}{c} x_{i}^{G+1}=\{\begin{array}{cc} u_{i}^{G+1} & if\;f\left(u_{i}^{G+1}\right)\leq f\left(x_{i}^{G}\right)\\ x_{i}^{G\;\;\;\;\;\;} & else \end{array} \end{array}$$ (13)
  Where, *f* represents the objective function, and $x_{i}^{G+1}$ is the *i-th* individual of the next population of generation G+1.

## 3 DE-based digital image watermarking

Two significant factors to evaluate the property of watermarking algorithm are invisibility and robustness. According to the features of discrete wavelet transformation, low frequency sub-band focus on the major energy of image, so embed watermark into low frequency sub-babd where the ability of algorithm against common attacks is improved, that is to say, the algorithm has a good robustness. However, high frequency sub-band represent the edge and texture information of an image. Embed watermark into high frequency sub-band will lead a better invisibility but it is easily affect with attacks, such as filtering and compression. Based on the above consideration, this article selects four sub-bands of a original image to embed watermark information respectively with the suitable scaling factors. The scaling factors are obtained by differential evolution algorithm, which insures the optimal algorithm properties and achieves a best balance between invisibility and robustness.

### 3.1 The scaling factor

Recently, the scaling factor of watermarking algorithm is all decided by experience, and usually set a constant. But in fact, the selection of factor value is adaptively determined by the difference of images, which DE algorithm can conveniently realize. Parameters that involved in DE algorithm primarily includes the size of population, mutation factor F, crossover factor CR and the vector solved the D-dimension question of algorithm. The processes of embedding and extraction of watermark information are implemented with right scaling factors which are achieved by a original image corresponding to the different frequency sub-bands. After watermark image is embedded into the original image, the algorithm applies some common attacks to the watermarked image. In line with varying degrees of distortion which suffered from various attack functions to images, combining with objective function involved in DE algorithm to make the optimization. The objective function has a close relationship with properties of watermarking algorithm invisibility and robustness, then the function *f* can be written as:

$$\begin{array}{c} Maximize\;f=NC\left(W,W^{*}\right)+NC\left(I,I^{*}\right) \end{array}$$ (14)

Where, W and W* denote watermark image and extracted watermark image, respectively. I, I* represent the original image and the watermarked image, respectively. NC (normalized correlation) reflects the similarity between two images, and it is an objective criteria to evaluate the effect of watermark extraction. A larger NC value means a higher degree of similarity between two images. The NC function is defined as follows,

$$\begin{array}{c} NC\left(X,\hat{X}\right)=\frac{\sum_{i}\sum_{j}X\left(i,j\right)\hat{X}\left(i,j\right)}{\sqrt{\sum_{i}\sum_{j}X{\left(i,j\right)}^{2}}\sqrt{\sum_{i}\sum_{j}\hat{X}{\left(i,j\right)}^{2}}} \end{array}$$ (15)

Where, X and $\hat{X}$ represent a original image (or watermark image) and watermarked image (or extracted watermark image) respectively.

### 3.2 Algorithm process

**Fig 4:**
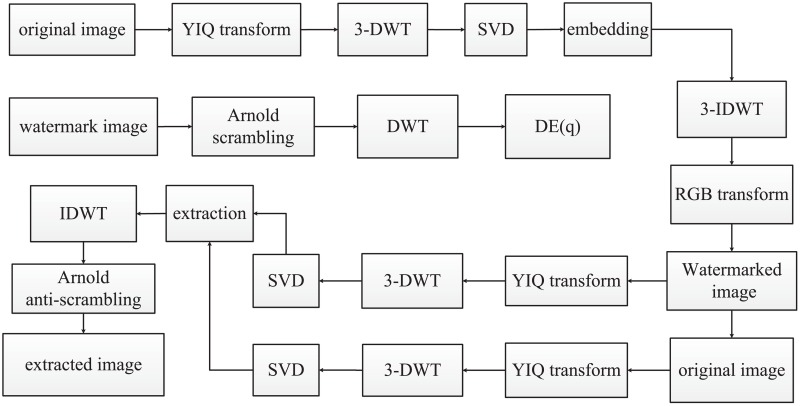
Flowchart of watermark embedding and extraction processes.

Generally, digital watermarking algorithm includes watermark embedding and extraction. In the process of watermark embedding, the scaling factor *q* is received by DE algorithm and the details of this operation is illustrated in Fig 4.

#### 3.2.1 Embedding process

1. Apply color space transform on a original image to change from the format RGB to YIQ, and extract the luminance component Y of image. Decomposed the component Y by the three-level DWT into four different frequency sub-bands a low frequency approximation sub-map LL and three high frequency approximation sub-maps LH, HL, HH.
2. Perform SVD operation on the four sub-bands which are obtained by previous step above, and get corresponding singular values *S*_*k*_.
  $$\begin{array}{c} Y_{k}=U_{k}S_{k}V_{k}^{T}\;\;\;\;k\in \left(LL,LH,HL,HH\right) \end{array}$$ (16)
3. Apply Arnold transform *τ* times on watermark image W to get the image W’, and then decompose W’ by performing one-level DWT to obtain LL (low frequency approximation sub-map), LH, HL, HH (three high frequency approximation sub-maps).
4. Modify the corresponding singular value of luminance component Y with the corresponding matrix of watermark image such that. Where the scaling factor *q* that is obtained by using DE algorithm.
  $$\begin{array}{c} S_{k}+q_{k}W_{k}^{{}^{\prime }}=C_{k}\;\;k\in \left(LL,LH,HL,HH\right) \end{array}$$ (17)
5. SVD operation is applied on matrix C to get three matrices *U*_*W*_, *S*_*W*_, $V_{W}^{T}$ as follows.
  $$\begin{array}{c} C_{k}=U_{wk}S_{wk}V_{wk}^{T}\;\;\;k\in \left(LL,LH,HL,HH\right) \end{array}$$ (18)
6. Compute the new values *S*_*wk*_ with the unitary matrices in the step 2 to apply anti-SVD operation such that.
  $$\begin{array}{c} U_{k}S_{wk}V_{wk}^{T}=Y_{wk}\;\;\;k\in \left(LL,LH,HL,HH\right) \end{array}$$ (19)
  After that, to obtain the luminance component $Y_{w}^{{}^{\prime }}$ of watermarked image, apply three-level IDWT process on it.
7. Combine $Y_{w}^{{}^{\prime }}$ with the YIQ format of the original image A and component Q together, then transform YIQ to RGB to get the watermarked color image *A*_*w*_.

#### 3.2.2 Extraction process

If $A_{w}^{*}$ represents the watermarked color image distorted by common attacks, extracted watermark image W* which is obtained by such steps as follows.

1. Apply the step 1 and 2 of the embedding process on the corrupted watermarked image $A_{w}^{*}$ to decompose into three matrices U*, $S_{w}^{*}$, V*^T^.
  $$\begin{array}{c} Y_{wk}^{*}=U_{k}^{*}S_{wk}^{*}V_{k}^{*T}\;\;\;k\in \left(LL,LH,HL,HH\right) \end{array}$$ (20)
2. Modify the corresponding singular values of image A’s luminance component Y with attacked matrix C* to get the matrix *W*′*, keeping the same value scaling factor *q* such that.
  $$\begin{array}{c} W_{k}^{{}^{\prime }*}=\left(C_{k}^{*}-S_{k}\right)/q_{k}\;\;\;k\in \left(LL,LH,HL,HH\right) \end{array}$$ (21)
3. One-level IDWT is applied on matrix *W*′* to obtain scrambled watermark image *W*′* which is attacked by certain common image processing.
4. Perform Arnold transform (*T* − *τ*) times, and T is the scrambling cycle, to get the extraction watermark image W*.

## 4 Experimental results and analysis

**Fig 5:**
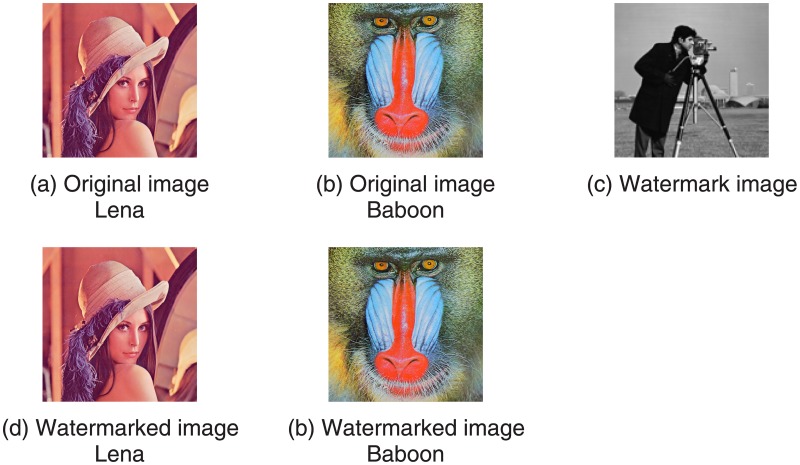
Images of watermarking embedding and extraction.

All experiments are implemented in MatlabR2013a environment, and we take two color images “Lena” and “Baboon” of size 512*512 as cover images and take gray image “cameraman” of size 128*128 as the watermark image, they are shown in Fig 5. Relative parameters used in differential evolution algorithm are set up according to the experiment results. Associated parameter settings are, NP = 150, F = 0.5, CR = 0.5 and D-dimension 4. In order to evaluate the robustness of this new algorithm, watermarked image is distorted by taking some various common attacks, such as a. Gaussian noise (GN), b. Salt and Pepper noise (SPN), c. rotation (RT), d. crop (CR), e. translation (TS), f. JPEG compression (JPEG), which are listed in Table 1 with their corresponding parameters of attacks. Where abbreviations of LU, RU, LM, RM, LD, RD respectively donate the position of top left, top right, left middle, right middle, bottom left and bottom right corner.

**Table 1:** List of various attacking operations and their parameter values.

No.	Attack Index	Parameter	Parameter Values
a	GN	Mean	0.01, 0.02, 0.03, 0.05, 0.07, 0.1, 0.2, 0.3
b	SPN	Mean	0.01, 0.02, 0.03, 0.05, 0.07, 0.1, 0.2, 0.3
c	RT	Angle	−45, −30, −15, −5, 5, 15, 30, 45
d	CR	Proportion	LU1/8, RU1/8, LM1/8, RM1/8, LU1/4, RD1/4, LD1/4, RU1/4
e	TS	Displacement	-(80×80), -(50×50), -(20×20), -(10×10), 10×10, 20×20, 50×50, 80×80
f	JPEG	Proportion	30%, 40%, 50%, 60%, 70%, 80%, 90%, 100%

### 4.1 Invisibility analysis

The PSNR (Peak Signal Noise Ratio) (21) metric is used to evaluate the quality of watermarked that is given as follows.

$$\begin{array}{c} PSNR=10log_{10}\left(\frac{{\left(X_{max}\right)}^{2}}{\left(1/n\times n\right)\sum_{i}\sum_{j}{\left(X\left(i,j\right)-\hat{X}\left(i,j\right)\right)}^{2}}\right) \end{array}$$ (22)

Where, *X* and $\hat{X}$ stand for the original image and the watermarked image; *X*_*MAX*_ is the maximum possible value of the image *X*; and the height or weight of the image is *n*. A larger PSNR value means the watermarking algorithm acquires a better invisibility. Generally, according to human visual identification rate, human eye is invisible to the modification of the image when PSNR>30. In other words, it is difficult to distinguish the difference between the original image and the watermarked image. Results are listed in Table 2, which illustrates the PSNR value of the original image and the watermarked image. As shown in Table 2, PSNR values are all above 40, indicating that our new algorithm has a high visual quality.

**Table 2:** PSNR values of the original image and the watermarked image.

No.	Attack index	PSNR value(Lena)	PSNR value(Baboon)
1	GN(0.02)	50.2849	53.7963
2	SPN(0.05)	44.1288	56.5509
3	CR1(LU1/4)	68.9824	51.3377
4	CR2(RD1/4)	46.6858	53.3866
5	RT1(150)	47.6478	50.9938
6	RT2(450)	51.2513	57.8768
7	JPEG1(10%)	46.6858	54.6147
8	JPEG2(15%)	58.2483	51.3849
9	JPEG3(75%)	50.4360	55.3521
10	JPEG4(80%)	46.9279	51.0517
11	TS1(80×80)	50.7108	65.5389
12	TS2(-(80×80))	63.5818	59.1615

### 4.2 Robustness analysis

**Fig 6:**
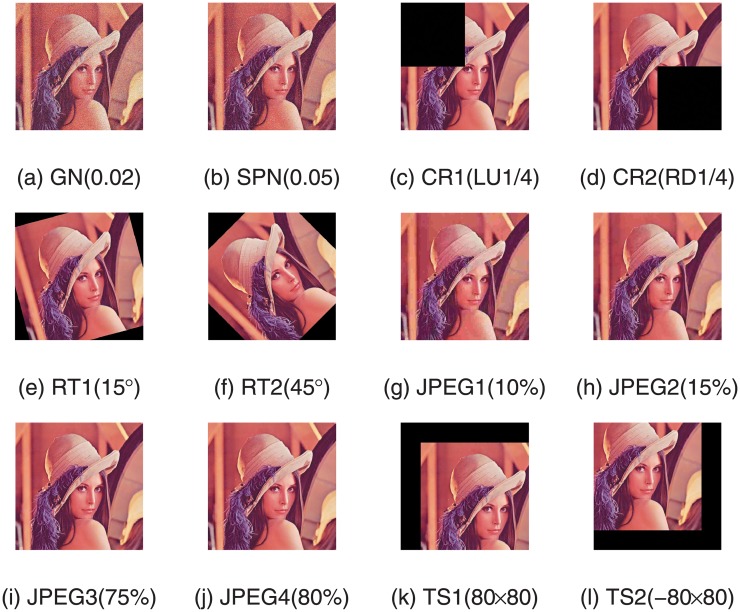
Watermarked images under various attacks.

**Fig 7:**
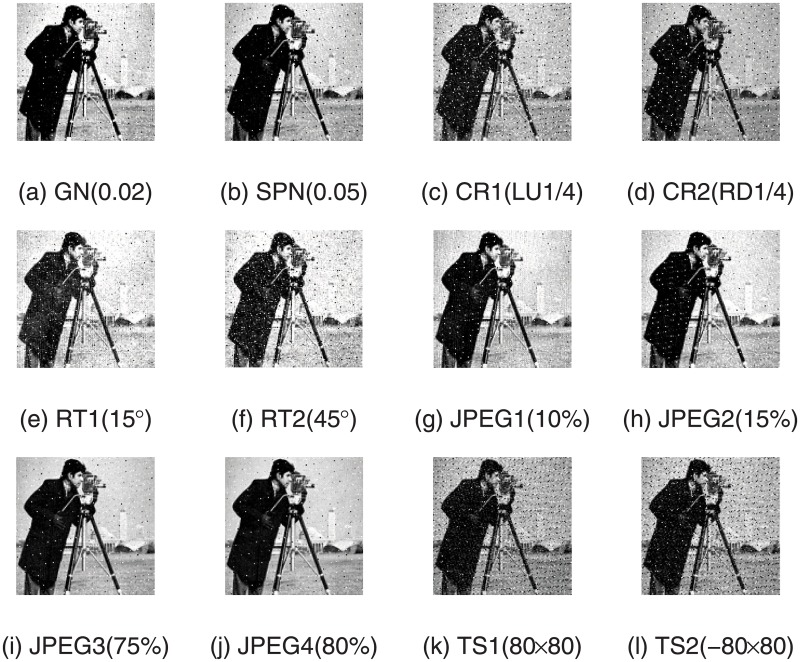
Extracted watermark images from distorted watermarked images.

**Fig 8:**
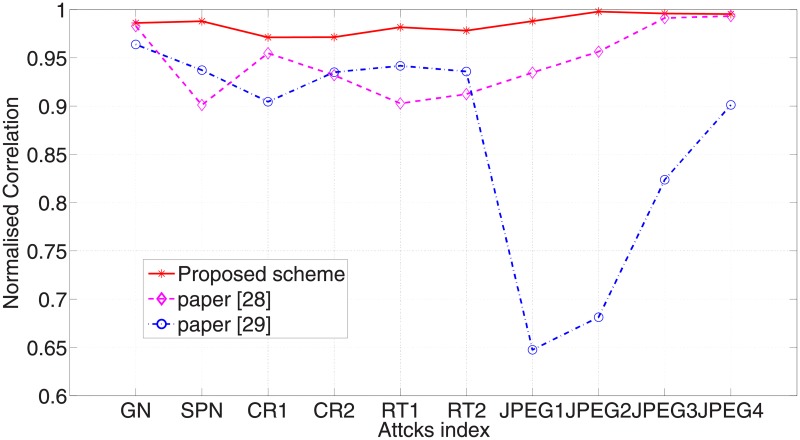
Comparison of algorithm in term of NC values.

**Fig 9:**
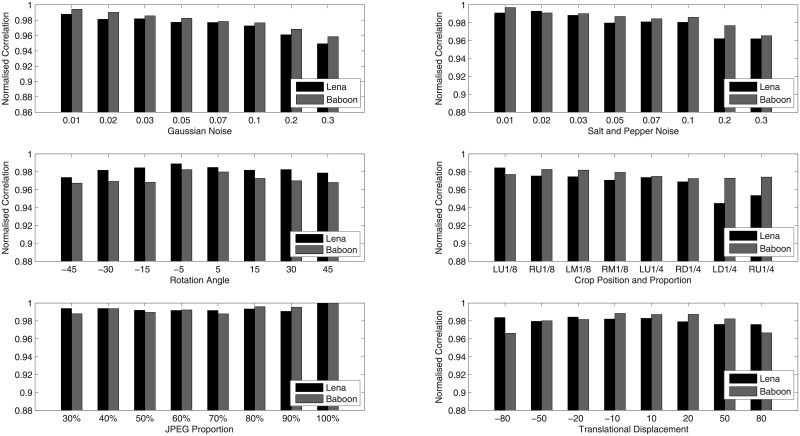
NC Values of watermark image under different attack operations.

The robustness reflects the ability of watermark system resisting various intention or accidental attacks. To estimate the robustness of our designed algorithm, the watermarked image is exposed to 12 various attacks. Take Lena as an example, the watermarked images after suffering various attacks are shown in Fig 6 while the extracted watermark images from them are shown in Fig 7. Intuitively from Fig 7, the extracted watermark images is clear, which is very similar to the original watermark image. Thus the robustness of our new algorithm is proved. In order to further evaluate the robustness of the proposed watermarking algorithm, we also compared our scheme with the previous algorithms in [28][29]. The scaling factors in [28] are set up in advance manually and the scheme in [29] is based image normalization. The NC values of watermark image and extracted watermark image are shown in Fig 8, which describes the difference between the proposed scheme with paper [28] and [29]. From the experimental results, we can see that NC values in the proposed algorithm are larger than the comparative literature and beyond 0.97, except the one corresponding to Gaussian noise attack. It is indicated that the watermarking algorithm based on DE algorithm has a better robustness than the constant scaling factor technique in the process of embedding watermark information. Furthermore, various attacks with different parameters are applied on watermarked images, and the corresponding NC values are shown in Fig 9. It is observed from the following figures that the proposed algorithm has a small variance of NC values in all the cases, which means the algorithm is robust to most of the attacks. Moreover, when the ratio of JPEG compression attack is 100%, the extracted watermark image is the same as the original watermark image. That is to say, this proposed algorithm can extract the original watermark image without distortion.

## 5 Conclusion

In this paper, we proposed a color watermarking algorithm based on differential evolution algorithm in wavelet domain. In our new algorithm, DE was performed to select scaling factors adaptively, which are used to embed watermark information and balance the conflict between the invisibility and robustness. Moreover, the proposed algorithm performs arnold transform for randomizing the watermark image. The experimental results show that the proposed watermarking algorithm has a high level of security. Meanwhile, it performs better in terms of invisibility and robustness. The future work includes introducing optimal methods in order to reduce the time complexity while enhancing robustness.
